# A New, Extremely Sensitive, Turn-Off Optical Sensor Utilizing Schiff Base for Fast Detection of Cu(II)

**DOI:** 10.3390/bios13030359

**Published:** 2023-03-08

**Authors:** Lotfi M. Aroua, Reham Ali, Abuzar E. A. E. Albadri, Sabri Messaoudi, Fahad M. Alminderej, Sayed M. Saleh

**Affiliations:** 1 Department of Chemistry, College of Science, Qassim University, Buraidah 51452, Saudi Arabia; lm.aroua@qu.edu.sa (L.M.A.); re.ali@qu.edu.sa (R.A.); aa.albadri@qu.edu.sa (A.E.A.E.A.); s.messaoudi@qu.edu.sa (S.M.); f.alminderej@qu.edu.sa (F.M.A.); 2 Laboratory of Structural Organic Chemistry-Synthesis and Physicochemical Studies (LR99ES14), Department of Chemistry, Faculty of Sciences of Tunis, University of Tunis El Manar, Tunis 2092, Tunisia; 3 Faculty of Sciences of Bizerte, Carthage University, Jarzouna, Bizerte 7021, Tunisia; 4 Chemistry Department, Faculty of Science, Suez University, Suez 43518, Egypt; 5 Chemistry Branch, Department of Science and Mathematics, Faculty of Petroleum and Mining Engineering, Suez University, Suez 43721, Egypt

**Keywords:** sensor, copper, fluorescence, synthesis, optical properties, chemical analysis

## Abstract

Throughout this research, a unique optical sensor for detecting one of the most dangerous heavy metal ions, Cu(II), was designed and developed. The (4-mercaptophenyl) iminomethylphenyl naphthalenyl carbamate (MNC) sensor probe was effectively prepared. The Schiff base of the sensor shows a “turn-off” state with excellent sensitivity to Cu(II) ions. This innovative fluorescent chemosensor possesses distinctive optical features with a substantial Stocks shift (about 114 nm). In addition, MNC has remarkable selectivity for Cu(II) relative to other cations. Density functional theory (DFT) and the time-dependent DFT (TDDFT) theoretical calculations were performed to examine Cu(II) chelation structures and associated electronic properties in solution, and the results indicate that the luminescence quenching in this complex is due to ICT. Chelation-quenched fluorescence is responsible for the internal charge transfer (ICT)-based selectivity of the MNC sensing molecule for Cu(II) ions. In a 1:9 (*v*/*v*) DMSO-HEPES buffer (20 mM, pH = 7.4) solution, Fluorescence and UV-Vis absorption of the MNC probe and Cu(II) ions were investigated. By utilizing a solution containing several metal ions, the interference of other metal ions was studied. This MNC molecule has outstanding selectivity and sensitivity, as well as a low LOD (1.45 nM). Consequently, these distinctive properties enable it to find the copper metal ions across an actual narrow dynamic range (0–1.2 M Cu(II)). The reversibility of the sensor was obtained by employing an EDTA as a powerful chelating agent.

## 1. Introduction

Copper Cu(II) ion is the third-most abundant metal ion needed by every living creature and is essential for several processes. In contrast, deficiencies or excesses of Cu(II) ions, as determined by cellular requirements, result in various diseases. Optical sensors with outstanding selectivity and sensitivity have been developed to detect Cu(II) ions. The designed sensor can detect luminescence in the refractive indices adsorption that arises from light and material interactions. They have garnered significant attention in recent years because of their simple and naked-eye identification, real-time recognition, cheap fee, excellent specificity against analytes, speedy response, and less equipment required in the investigation process. Copper (Cu(II)) ion is one of the essential metal ions. It plays a significant part in a variety of processes that are necessary to both nature and the human body [1,2].

As an enzymatic activator of oxidative enzymes involving tyrosinase, lysyl oxidase, cytochrome c oxidase, superoxide dismutase, and a number of metalloenzymes [3,4], it plays a vital function in the human body. It is an essential catalyst for iron absorption and ferroheme synthesis [5]. Under normal conditions, Cu(II) ion does not harm the human body; excess and deficiency can produce diverse effects. Although minimal levels of Cu(II) ion are necessary for normal physiological processes, exceeding the recommended daily consumption is harmful to human health [6,7]. The increase or decrease in copper ion concentration causes several disorders related to iron homeostasis, which is controlled by copper ions.

Excessive Cu(II) ion consumption can result in diarrhea, vomiting, vertigo, and abdominal pain [8]. Long-term exposure causes the accumulation of free Cu(II) ions in the body, which, owing to redox activity, produces reactive oxygen species that are harmful to fatty acids, DNA, and tissues. Oxygen radicals have the potential to be cytotoxic [9,10]. Several illnesses may emerge depending on the state of the body’s hemostasis. In addition to Wilson’s, Parkinson’s, Menkes, neurodegenerative, and Alzheimer’s illnesses [11,12], increasing the content of Cu(II) ion produces Huntington’s disease, and acute hepatic and renal failure [13,14]. Environmental Protection Agency (EPA) guidelines for Cu(II) ions in drinking water are 1.3 ppm [15,16]. The copper concentration in a healthy person’s blood serum should be 100–150 μg/dL [17,18]. Additionally, home, industrial, and agricultural operations emit Cu(II) ions into the environment [19]. Cu(II)-containing pesticides are often used to stimulate crop growth while also preventing illness. Since 1991, the EPA has limited the amount of Cu(II) ion in tap water to 20 μM [20]. In contrast, the World Health Organization (WHO) has set the limit at 30 μM [21]. The excessive use of these pesticides may contaminate different water resources.

Several techniques exist for detecting trace metals. Various advanced techniques are utilized in the sensing process of copper ions [22,23,24]. Nevertheless, these techniques have downsides. For example, many of these techniques are pricey and difficult, and important factors such as dependability, precision, and speed must be addressed [25,26]. It is imperative and urgent to enhance sensitive, simple, quick, repeatable, cheap, and analytical techniques for detecting Cu(II) ions in ecological and medicinal materials [27]. The detection mechanism relies on the unique binding of the specified analyte to the receptor. Based on the contact, the transducers may detect characteristics that include changes to the pH, electron, mass transfer, temperature, a change in optical qualities, and potential differences. This method turns the receptor’s response into a significant gesture exactly proportional to the presence or quantity of the analyte [28,29]. As a result of its low-priced, simple miniaturization, and rapid output of semi-quantitative data, sensor research has grown in popularity and importance in recent years. Because of this, sensors have become indispensable means for scientific ecological and dietary investigation, as well as the detection of chemical and biological contaminants [30,31,32,33].

The Schiff base is a magnificent class of target compounds owing to diverse applications in different fields that cover analytical, biological, and coordination chemistry. The Schiff base compound achieved enormous significance in the healthcare and medicinal industries and fascinated scientists to generate a novel construction outstanding variety of bioactivities involving antimicrobial [34], antibacterial [35], antifungal [36], anti-ulcer [37], anticonvulsant [38], antioxidant [39], anti-inflammatory [40], analgesic [41], and anti-tubercular [42]. As a part of biological activities, Schiff base emerged as a key intermediate to elaborate new structures that find multiple applications as optical [43], photosensitizes in dye-sensitized solar cells [44], catalyst [45], polymers stabilizers [46], semiconducting multicolumnar mesophases [47], pigments [48], dyes [49], sensors [50,51,52,53] and luminescence [54]. Carbamate derivatives are one of the prospective target molecules utilized in producing pharmaceuticals, cosmetics, pesticides, and polyurethanes [55]. Interestingly, naringenin carbamate and chalcone-O-carbamate have been studied as multifaceted Alzheimer’s disease treatments [56,57]. 

In this study, we describe a switch turn-OFF Schiff base sensor probe. Our group developed a practical, extremely selective, reversible, and susceptible chemosensor with a low detection limit and excellent sensitivity for copper metal ions. Moreover, the copper ion detecting process is based on the ICT mechanism. The Schiff base chelating ligand was produced following the scientific literature. The synthesized ligand is a unique optical sensor for detecting copper ions. The detection technique relies on measuring the fluorescence increase of the Schiff base sensing molecule throughout the complexation process based on Cu(II).

## 2. Experimental

### 2.1. Materials and Methods

All compounds were purchased from (Sigma-Aldrich, St. Louis, MO, USA) and utilized with no additional processing. The absorbance spectra (UV–vis) were collected using a 1 cm quartz cell and an Evolution^TM^ 200 series UV-Visible spectrophotometer. For emission and excitation spectrum investigations, a 1 cm quartz cell was used with a JASCOFP6300 spectrofluorometer to detect fluorescence spectra. The 20 mM HEBES buffer stock solution with a pH of 7.4 has been made. On a Bruker NMR operating at 800 and 125 MHz, the ^1^H and ^13^C-NMR spectra were acquired. The chemical shifts were calculated using DMSO-d6 as the solvent and TMS as the standard internal reference. The melting points were obtained using the Stuart SMP30 equipment. For analytical TLC, Silica Gel 60 F254 plates (40–60 m) were used from Sigma, and the chromatogram was produced using a 254 nm UV lamp. All reactions were conducted in a nitrogen-protected dry environment using oven-dried glassware without additional purification.

### 2.2. Procedure for MNC Synthesis 

In a 100 mL round bottom flask, a solution of 4-mercaptoaniline (1.25 g, 10 mmol) in 15 mL of dichloromethane was added to a solution of salicylaldehyde (1.22 g, 10 mmol) in 40 mL of dichloromethane. After 2 h of refluxing, the mixture was allowed to cool to room temperature. A 30 mL dichloromethane solution of naphthyl isocyanate (1.69 g, 10 mmol) was added and agitated for 30 min. The mixture was then heated under reflux for 3 h. In order to monitor the reaction progress, diethyl ether/hexane (70/30) was utilized as an eluent. Following cooling, the mixture was filtered, and the crude material was recrystallized from ethanol to yield (3.46 g, 87.4%). M.p. = 215–217 °C. ^1^H NMR (850 MHz, DMSO *d*_6_): 12.88 (s, 1H, NH), 10.53 (s, 1H), 9.00 (s, 1H, CH=N-), 8.04 (d, 1H, *J* = 8.5 Hz, H_arom_), 7.97 (d, 1H, *J* = 7.68 Hz, H_arom_), 7.85 (d, 1H, *J* = 7.65 Hz, H_arom_), 7.68 (dd, 1H, *J* = 7.65 Hz, *J* = 1.7 Hz, H_arom_), 7.63–7.57 (m, 5H, H_arom_), 7.52 (t, 1H, *J* = 8.5 Hz, H_arom_); 7.47 (m, 2H, H_arom_), 7.44 (m, 1H, H_arom_), 7.02 (m, 2H, H_arom_);^13^C NMR (212.5 MHz, DMSO-*d*_6_) *δ*(ppm): 164.6 (C=O), 160.7 (HS-C), 149.3, 136.8, 134.2, 134.08, 134.09, 128.6, 127.0, 126.7, 126.0, 123.2, 122.4, 119.79, 119.72, 117.1. IR (cm^−1^) *ν*: 3245 (NH), 3051(CH), 1650 (C(O)NH), 1614(C=N); 1568(C=C), 1519, 1492, 1481, 1455, 1275, 1240, 1182, 1172. The NMR and IR spectra were displayed in the supporting information (Figures S1 and S2).

### 2.3. Optical Measurements

For the different kinds of examined metal ions, such as Gd(III), Ag(I), etc., stock solutions of metal nitrates were prepared. Fluorometric titration was conducted with a free ligand concentration of 1.25 M in a buffer solution containing 1:9 (*v*/*v*) DMSO-HEPES (pH = 7.4). In a 1.0 M aqueous solution, the UV-Vis spectra of free MNC and complexes (MNC-Cu(II)) were assessed. Throughout Absorbance titration, it is reasonable to suppose that the overall volume of free ligand MNC and metal ions is 2.0 mL since the volume of metal ions can be disregarded compared to that of free MNC. The length of the excitation and emission slits within every experiment was 5 nm.

## 3. Results

### 3.1. MNC Probe Synthesis 

**Scheme 1:**
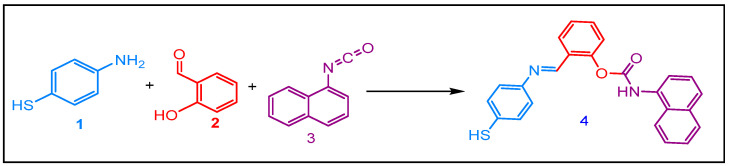
Synthesis of the Schiff Base carbamate.

Our approach started with synthesizing Schiff base carbamate via the condensation of equimolar amount 4-mercaptoaniline **1** and salicylaldehyde **2** in anhydrous boiling dichloromethane. After 3 h of contact, one equivalent of naphtylisocyanate **3** was added. The reaction afforded the corresponding Schiff base carbamate **4** at 85 % yield (Scheme 1). 

### 3.2. Optical Characteristics of MNC

**Figure 1:**
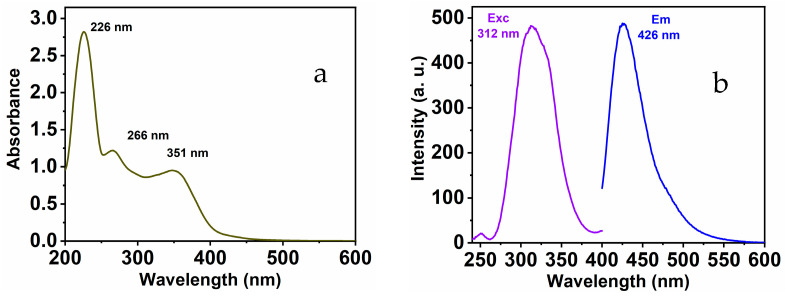
MNC (**a**) UV-Vis; (**b**) emission and excitation spectra.

The as-prepared MNC chemical sensor shows distinguishing optical characteristics; these features were collected in a 1:9 (*v*/*v*) DMSO-HEPES buffer solution (20 mM, pH = 7.4) (see Figure 1). Under excitation with 312 nm, the free chemical sensor displays a distinct peak located at 426 nm. In addition, the absorbance spectra provide the existence of three maximums at 226, 266, and 351 nm correspondingly. These peaks might be ascribed to π-π* and n-π* transitions.

**Figure 2:**
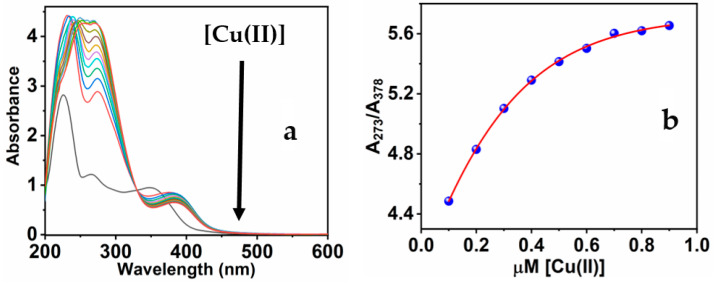
(**a**) The UV-Vis spectra of MNC throughout the gradual adding Cu(II); (**b**) the relation of the band ratios at 273 and 378 nm (A_273_/A_378_) against [Cu(II)].

Absorbance and fluorescence methods were used to observe the fluorescence response of MNC to the copper ions. In addition to the two notable absorbance peaks of the free MNC probe, the addition of Cu(II) ions to the MNC molecules formed a novel absorbance peak at 378 nm [58]. The absorbance of the 378 nm peaks quenches when more copper ions are added. In contrast, the 266 absorption peak begins to intensify and shift to 273 nm, a redshift, when Cu(II) is added to the MNC solution. With the development of an isosbestic point at 329 nm, the synchronized behavior of the three absorption peaks continues (Figure 2a).

Intriguingly, the peculiar response of the MNC UV-Vis spectra verifies the binding procedure involving Cu(II) metal and the functioning groups, which include oxygen and nitrogen atoms of the sensitive chemical sensor [59]. The association between the absorption ratio at 273 and 378 nm of the chemical sensor and as a function of Cu(II) concentration throughout the range of 0.1 to 0.9 M is noteworthy (Figure 2b). Particularly, when the Cu(II) concentration reaches an equal molar ratio of 1:1 (MNC/Cu(II)), the absorbance starts to be constant even at higher molar ratios. The ratiometric detection approach demonstrates that the mechanism of Cu(II) and MNC interaction is a ratio of 1:1 stoichiometric.

**Figure 3:**
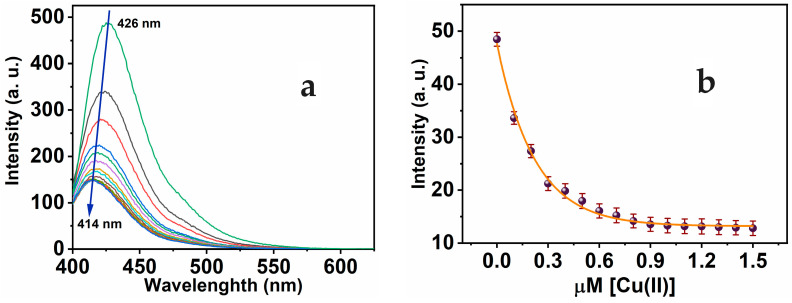
(**a**) Luminescence of MNC 10 μM in a 1:9 (*v*/*v*) DMSO-HEPES buffer (20 mM, pH = 7.4) during gradual adding Cu(II) of 0–1.5 μM range, λ_exc_ 312 nm; (**b**) calibration curve of MNC intensity versus [Cu(II)].

Furthermore, the MNC sensing molecule exhibits luminescent properties, through the dominant peak showing at 426 nm and λ_exc_ at 312 nm. The optical characteristics of MNC demonstrate the lack of overlap seen between absorbance and luminescence, and a considerable Stocks shift (Δλ = 114 nm) was observed. The substantial Stokes shift reduces self-quenching, which is necessary for potential implementation. With the stepwise addition of Cu(II) to MNC, the luminescence sensitivity of the prominent peak at 426 nm was substantially quenched. As seen in Figure 3a, the amplitude of signal attenuation reached about 74.43%. Moreover, the addition of Cu(II) ion to the MNC probe in a concentration range from 0 to 1 equivalent of Cu(II) causes the prominent emission peak to quench and shift to 414 nm. However, the fluorescence intensities stabilized following the addition of more than 1 equivalent of Cu(II) ions. The fluorescence titration demonstrated a nonlinear fit of the fluorescence emission titration curve. In accordance with absorption titration results, a 1:1 complex ratio was observed between the MNC chemical sensor and Cu(II). 

### 3.3. PH Influence on the MNC Luminescence 

**Figure 4:**
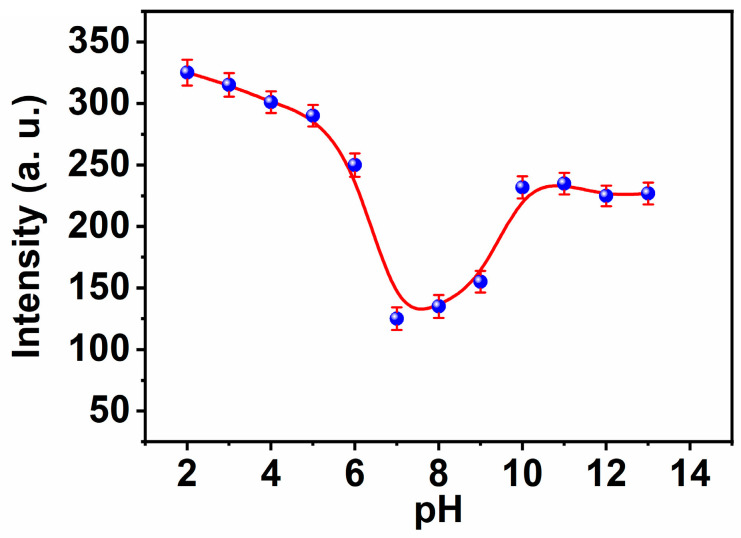
The influence of the pH on the fluorescence intensities of MNC ligand under λ_exc_ 312 nm.

The majority of metal cations detection depends on optical sensor fluorescence modification coupled with hydrogen proton transfer. Changes in the pH of the ambient solution have an effect on this occurrence. In the pH range of 2 to 11, the effect of pH on the selectivity and responsiveness of the chemical sensor to copper ions was examined. By adjusting the pH of the aqueous solution with 20 M HEPES buffer solutions at a certain concentration of Cu(II) ion (1 μM) and excitation at 312 nm, we were able to determine the Cu(II) concentration (Figure 4). As seen, in the pH range of 2.0 to 7.0, the chemical sensor’s response is proportional to pH because the Schiff base exists in protonated form without complex formation. The binding of the proton to the nitrogen and oxygen atoms of the active groups inhibits the formation of complexes to a certain degree. However, the observed increase in MNC sensor fluorescence in response to alkaline pH or high pH > 7 is owing to the precipitation of Cu(II) hydroxide from the aqueous solution. In a variety of pH 7.4 buffered systems, including the HEBES buffer, the high reactivity and specificity of the MNC sensing molecule for copper ions were investigated.

### 3.4. Quenching Phenomenon 

Collisional quenching of fluorescence is described by the Stern-Volmer equation [60]:

$$\frac{F_{0}}{F}=1+K_{sv}\left[Q\right]$$

**Figure 5:**
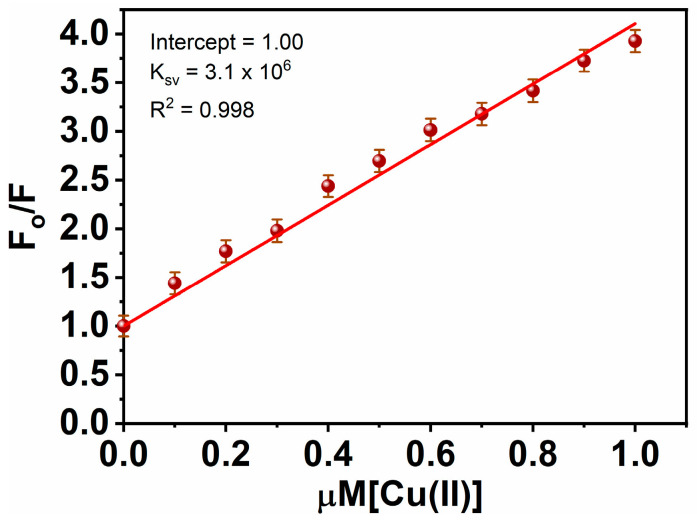
Stern Volmer plot for Cu(II)-MNC.

F_0_ and F are the fluorescence intensities in the absence of a quencher and its presence, respectively, correspondingly, in this equation. K_sv_ is the quenching constant, and Q is the quencher concentration. K_sv_ denotes the Stern-Volmer quenching constant. Typically, data on quenching are displayed as graphs of F_0_/F vs. [Q] (see Figure 5). A linear Stern-Volmer curve demonstrates that collisional fluorescence quenching has occurred. Over a broad range from 0 to 1 M, R^2^ = 0.998% demonstrated an exceptional linear dependency. In order to assess the nanosensor’s sensitivity, the limit of detection (LOD) was estimated to be 1.45 nM, supposing that emission intensity can be assessed with 1% accuracy [61]. Notably, these LODs are far lower than the Cu^2+^ content (31.5 μM) recommended by the WHO for drinking water characteristic guidelines [62]. Furthermore, the LOD of the MNC is compared to some of the recently reported sensors (Table 1).

**Figure 6:**
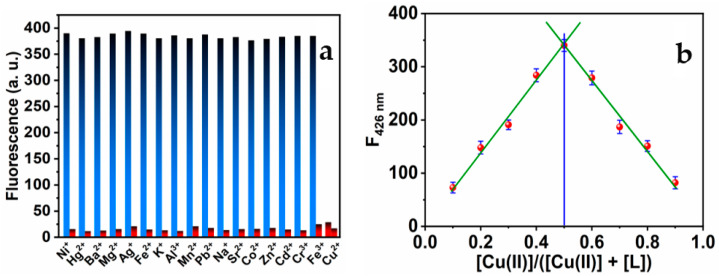
(**a**) Maximum luminescence intensities of the MNC sensing molecule (I) in the presence of various metal ions (blue bar) and (II) in the presence of the same metal ions and Cu(II) ions (red bar). The ratio of (MNC: Cu(II): M(II) has a 1:1:1 ratio (λ_ex_ = 312 nm); (**b**) Job’s plot of MNC with Cu(II) ions.

**Figure 7:**
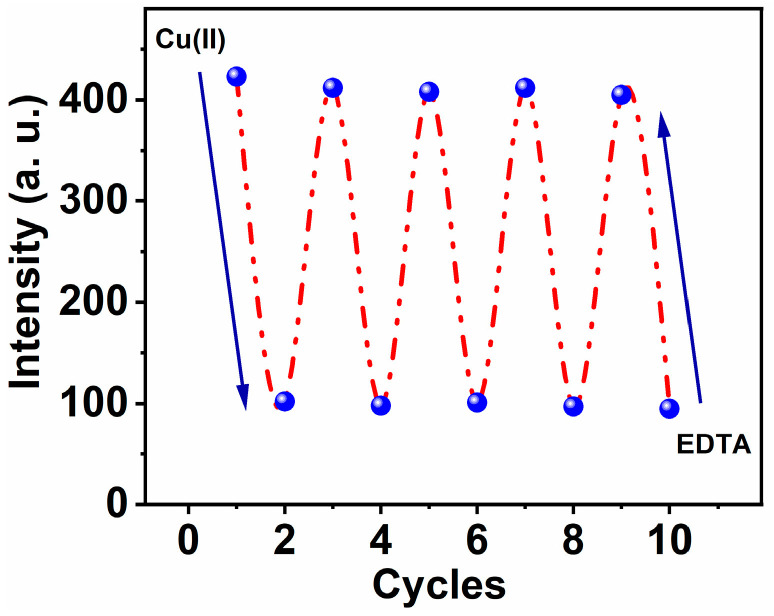
Reversibility of MNC-Cu(II) and EDTA mutually, λ_ex_ = 312 nm and λ_em_ = 426 nm, respectively.

Under the optimum conditions, the interaction between MNC molecules and Cu(II) was investigated in the absence and the presence of various metal ions of a 1 μM concentration. There were minor variations in the MNC probe’s emission intensities. As seen in Figure 6a, none of the studied interfering salt ions had any discernible effect on the chemical detection of Cu(II) ions. Consequently, the chemical sensor might be used as a highly selective sensor for Cu(II). The MNC:Cu(II) complex stoichiometric ratio was established using Job’s plot-based luminescence analyses [69,70]. The Job’s plot (see Figure 7b) was generated by varying the copper ion concentration within a range of up to 1 M. The greatest fluorescence intensity quenching value was observed when the molar ratio of Cu(II) was around 0.5, indicating that MNC and Cu(II) ions formed a 1:1 complex. In addition, this is consistent with the Absorbance titration.

### 3.5. Reversibility 

Ethylenediaminetetraacetate (EDTA), a very effective binding reagent for especially Cu(II) ions, was used to investigate the reversibility of the MNC-based optical chemosensor. Cu(II)-MNC complex solution, with a molar ratio of (1:1) Cu(II) to MNC and a concentration of 10 μM, was exposed and mixed with an equal molar concentration of EDTA solution. Upon addition of EDTA, their molecules begin to swap MNC molecules, and an immediate Cu-EDTA complex formed. Intriguingly, the exchange chelation procedure was followed by an increase in fluorescence caused by the freed chemical probe MNC. Consequently, the fluorescence intensity increased until it reached roughly 75% of the intensity of the free form (see Figure 7).

### 3.6. Quantum Yield

The quantum yield (QY) estimate for MNC was presented as 0.22. The QY was detected depending on a standard and reference chromophore of quinine sulfate [71]. The aqueous solution of the reference chromophore in sulfuric acid was prepared to give a 55% QY. The ultimate deviation for such QY was predicted to be (±6%).

$$Q_{X}=Q_{R\;}\frac{A_{R}\;I_{s}n_{s}^{2}}{A_{X}\;I_{R}\;n_{s}^{2}}$$

While

- X and R point to the MNC and reference solutions
- n is a refractive index at room temperature
- I is the integrated area under the peak
- A is the maximum absorbance peak.

### 3.7. Binding Constant K_b_

**Figure 8:**
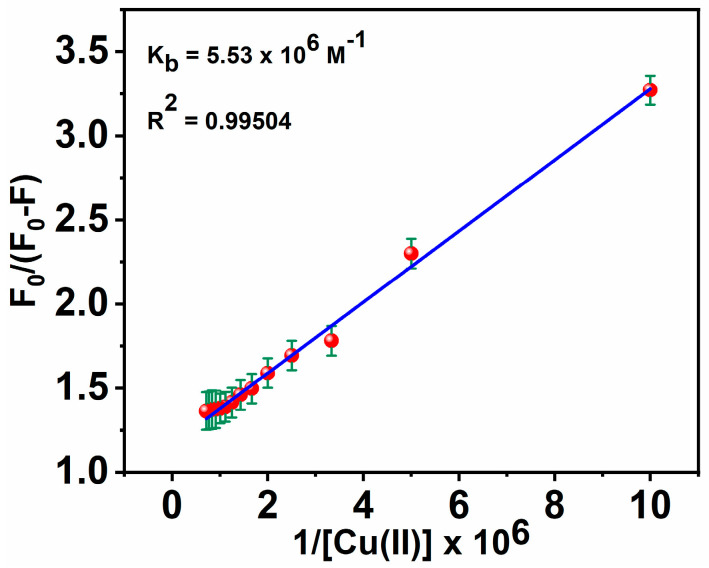
Stern-Volmer plot for calculating K_b_ of MNC-Cu(II).

To further comprehend the metal ligand binding mechanism, variations in the fluorescence spectra of the MNC probe in the presence of various Cu(II) concentrations were examined to calculate the binding constant. On the basis of a 1:1 stoichiometry, the binding constant was calculated using the modified Stern-Volmer equation,

$$\frac{F_{0}}{F_{0}-F}=\frac{1}{A}+\frac{1}{A.\;K_{b\;}.\left[Q\right]}$$

where F_0_ is the fluorescence intensity of the free ligand MNC, F is the fluorescence intensity of the MNC-Cu(II) complex, Q is [Cu(II)], A is a constant, and K_b_ is a binding constant [72]. When F_0_/(F_0_ − F) was plotted as a function of the 1/[Q] concentration, a linear relationship was obtained: (y = α + βx), y = F_0_/(F_0_ − F), α (intercept) = 1/A, β (slope) = 1/A.K_b_, x = 1/[Q], and K was estimated from α/β (Figure 8). Inferred from the fluorescence titration curves of the MNC probe with Cu(II), K_b_, the binding association constant, was calculated to be 5.53 × 10^6^ M^−1^.

**Figure 9:**
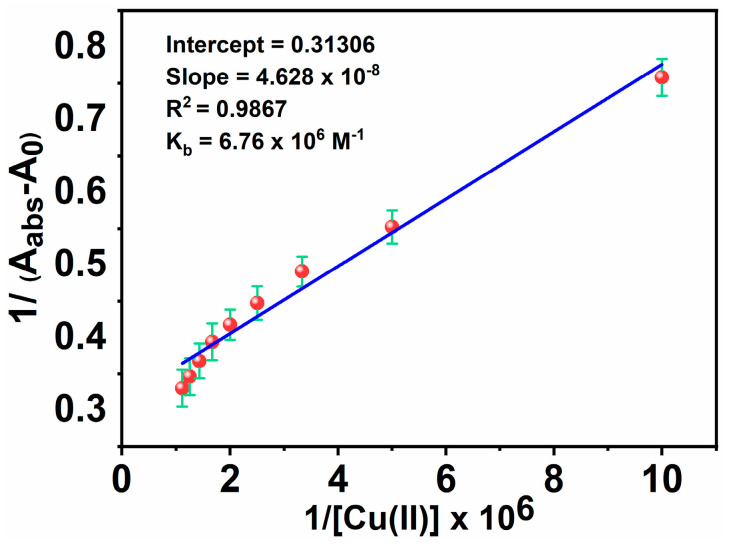
Benesi-Hildebrand plots for K_b_ of MNC- Cu(II).

Also, the extent of the binding of MNC to Cu(II) ions is determined based on the absorbance enhancement at 266 nm using an experimental plot of absorption data based on the Benesi-Hildebrand relation [73]:

$$\frac{1}{A_{abs}-A_{0}}=\frac{1}{A_{C}-A_{0}}+\frac{1}{\left(A_{C}-A_{0}\right).\;K_{b\;}.\left[Cu\left(II\right)\right]}$$

where A_0_, A_abs_, and A_c_ are the absorption values in the absence of, at the intermediate, and at the saturation of Cu(II) ion interaction, respectively, and [Cu(II)] indicates the concentration of Cu(II) ion added. By linearly fitting an absorbance titration curve, the binding constant (K_b_) was calculated (Figure 9). The greater value of the binding constant (6.76 × 10^6^ M^−1^) suggests that the interaction between MNC and Cu(II) ion is strong.

### 3.8. Theoretical Study

Software Gaussian09 conducted mathematical methods that rely on the density functional theory (DFT) in conjunction with the time-dependent DFT (TDDFT) [74]. The purpose is to clarify the complex structure of Cu^2+^ and its electronic properties, and It may lead to fluorescence increase or decrease. The structures’ geometry was developed using the module CAM-B3LYP, and the basis set was 6–31G(d)/Lanl2dz. The LANL2DZ basis set treated Cu, and we used a 6–31G(d) basis set for all the other atoms. At the same level of theory, vibrational frequencies were calculated to confirm that all the structures are stable. As the experiment is realized in methanol, TDDFT with SMD solvation model and methanol was used on the compounds optimized by CAM-B3LYP at the PBE1PBE/Lanl2DZ/6–31 + G(d) level of theory.

**Figure 10:**
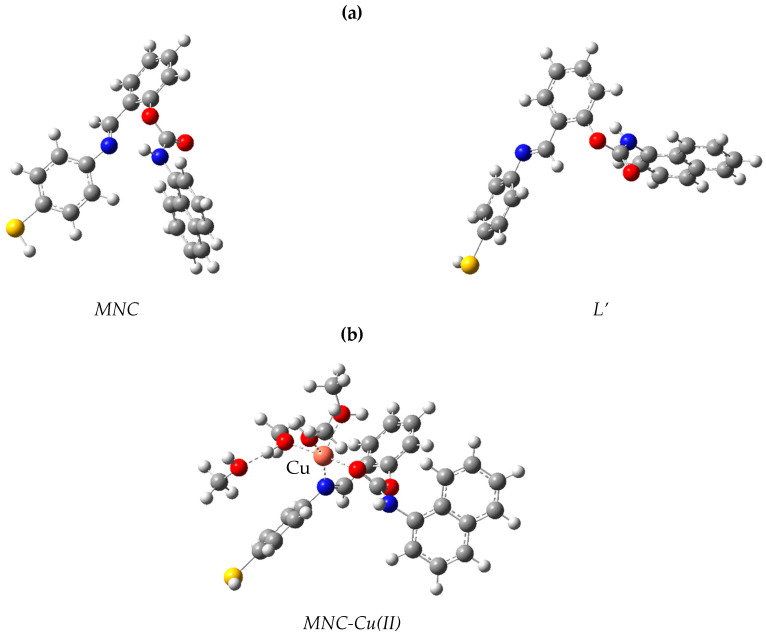
Model structures for (**a**) MNC and (**b**) MNC-Cu(II).

**Figure 11:**
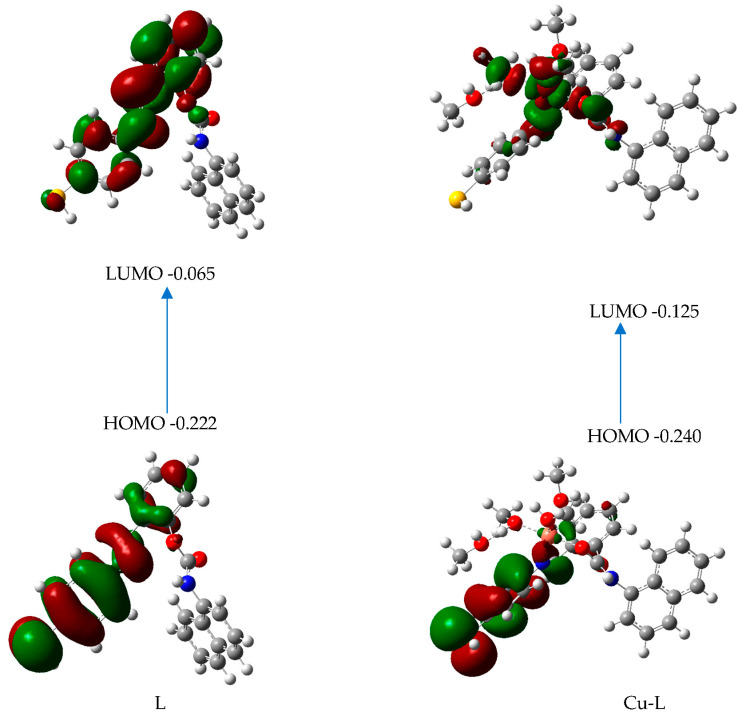
Frontier orbitals and their energies for L and Cu-L.

Ligand conformers for L and L’ are presented in Figure 10a. The L conformer is more stable than the L’ structure by 3.31 kcal/mol (Table 2). The theoretical study of the ligand’s electronic structure alone will focus on the most stable conformer, L. The copper complex optimized structure (Cu-L) is represented in Figure 10b. In this figure, we see that the copper center in the complex is five coordinated to the ligand L through one nitrogen atom and one oxygen atom. The bond length of Cu-N is 2.032 Å, and the bond length of Cu-O is 1.95 Å in the optimized structure. Thus, the ligand L can accommodate the cation Cu^2+^ by forming bonds with one nitrogen and one oxygen. The likely transitions in such chromophores were identified using time-dependent DFT Numerical simulations. Figure 11 depicts the border molecular orbitals of MNC and Cu-LMNC-Cu(II). The data of electronic transitions, wavelength, and oscillator strength from TDDFT calculations are found in Table 3.

The electron density for the frontier orbitals in the ligand alone is almost located on the sulfur atom and the two aromatic rings linked by a CN double bond. In L-Cu, the delocalization of electrons between the HOMO and LUMO transition levels is due to ICT: the electron density in HOMO is almost on the sulfur atom and the two aromatic rings linked by a CN double bond, but in the LUMO, it is almost located on the metal and the atoms of the ligand linked to the metal. We might speculate that the intensity quenching in this molecule is the consequence of MNC-Cu(II) binding. The weak value of the oscillator strength for this transition in the complex, which is equal to 0.0156 (Table 2), and the relatively large oscillator strength for the transition in MNC, which is equal to 0.4950 (Table 3), support these findings.

## 4. Conclusions

In summary, an extremely sensitive and innovative turn-off chemical sensor probe MNC was developed being prepared for the sensing of Cu(II) ions depending on a chelation approach to avoid interfering with different types of metal ions in a 1:9 (*v*/*v*) DMSO-HEPES buffer (20 mM, pH = 7.4) solution. The colorimetric and absorption MNC sensor revealed a considerable Stocks shift of 114 nm; thus, the proposed chemosensor provides a novel approach for creating a chemical probe with substantial luminescence changes. The MNC sensor can readily detect Cu(II) ions with high selectivity, exceptional sensitivity, and a detection limit (LOD) of 1.45 nM. Copper ions and MNC in nano concentrations exhibited an excellent linear relationship that could be used for quantitatively measuring Cu(II) ions. Job’s experiments demonstrated that MNC chelates Cu(II) ions in a 1:1 metal/ligand ratio. The studied chemical sensor depends on the binding of Cu(II) to the MNC synthetic chromophore and the formation of the MNC-Cu(II) complex molecule for its mechanism. DFT and TDDFT demonstrate that the quenching of emission intensities in this synthetic MNC molecule results from ICT throughout MNC to the binding metal ions. This chemical sensor based on an MNC sensor might provide a unique method for detecting Cu(II) in the environment and for research purposes.

## Figures and Tables

**Table 1:** Comparison of Cu(II) optical sensors found in the literature with the investigated work.

Research Group	LOD	Mechanism	Ref.
Torawane et al.	0.35 μM	Quenching	[63]
Tang et al.	0.48 μM	Quenching	[64]
Yang et al.	0.49 μM	Quenching	[65]
Chou et al.	0.87 μM	Enhancement	[66]
Park et al.	2.90 μM	Enhancement	[67]
Ghosh et al.	1.01 μM	Red shifting	[68]
Saleh et al.	0.011 μM	Quenching	[7]
This work	1.45 nM	Quenching	-

**Table 2:** Calculated Relative Energies of the ligands.

Complex	Relative Energy (kcal/mol)
L	0
L’	+3.31

**Table 3:** TDDFT outcomes of certain transitions, including associated absorbance energies and oscillator strengths.

Complex	Transition	λ (nm)	Oscillator Strength
L	HOMO -> LUMO	353	0.4950
L-Cu	HOMO(b) -> LUMO(b)	521	0.0156

## Data Availability

Not applicable.

## Supplementary Materials

The following supporting information can be downloaded at: https://www.mdpi.com/article/10.3390/bios13030359/s1, Figure S1: FTIR spectra of the MNC probe; Figure S2: NMR spectra of the MNC probe.
