# Supplementary material for: A New, Extremely Sensitive, Turn-Off Optical Sensor Utilizing Schiff Base for Fast Detection of Cu(II)
Source: Biosensors (Basel). 2023 Mar 8;13(3):359. doi: 10.3390/bios13030359 (PMC10046006; doi:10.3390/bios13030359)
Supplement: Supplementary file 1 [file biosensors-13-00359-s001.zip › biosensors-2238012-supplementary.pdf]

Supporting Information

A new, extremely sensitive, turn-off optical sensor utilizing Schiff base for fast detection of Cu(II)

Lotfi M. Aroua [1,2,3], Reham Ali [1,4], Abuzar E.A.E. Albadri [1], Sabri Messaoudi [1,3], Fahad M. Alminderej [1] and Sayed M. Saleh [1,5,*]

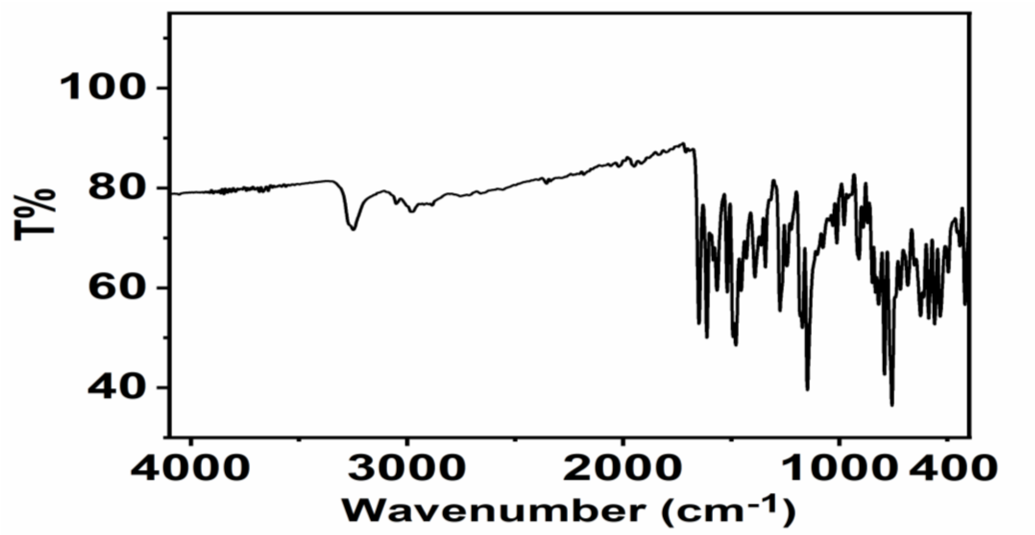

Figure S1. FTIR spectra of the MNC probe

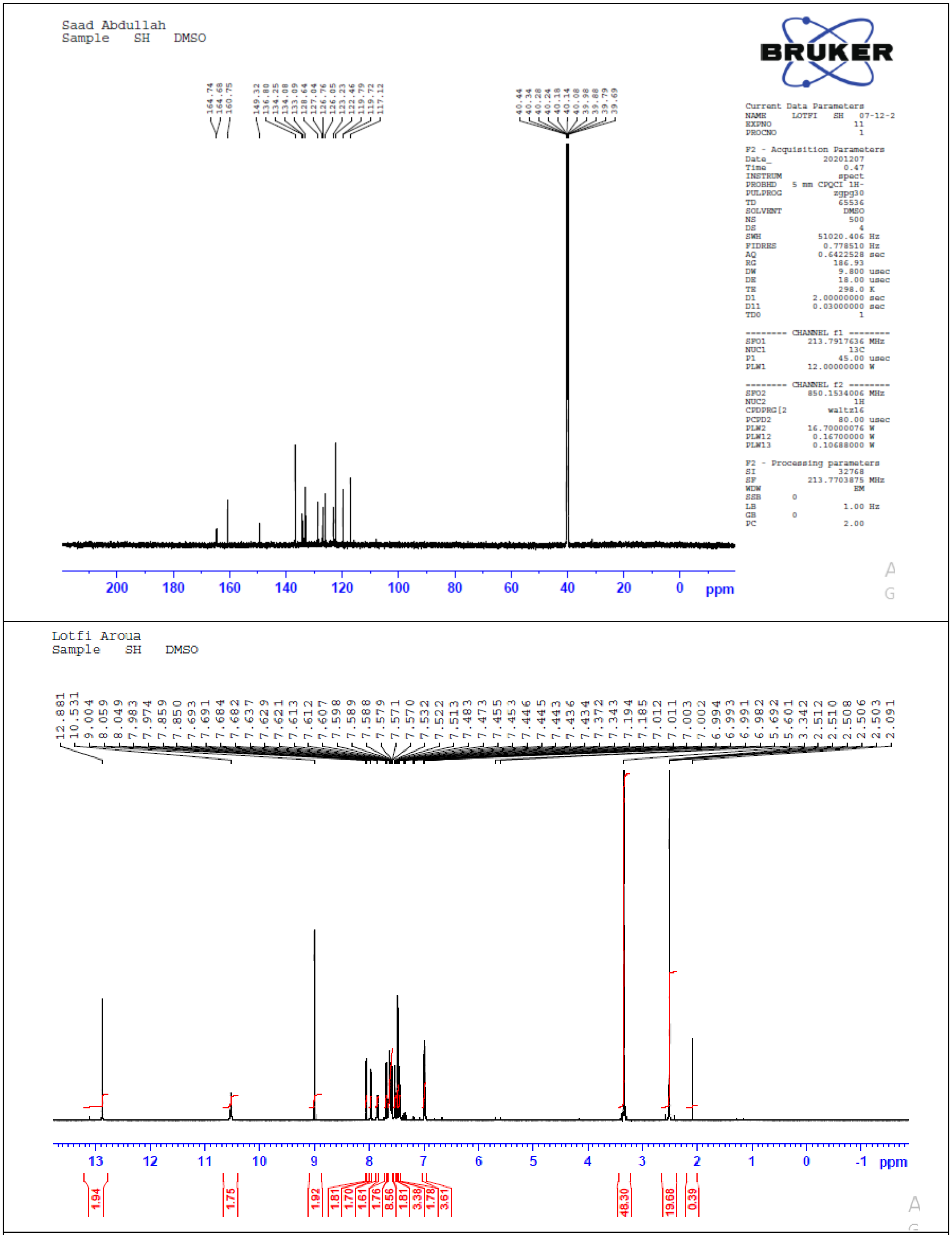

Figure S2. NMR spectra of the MNC probe
